# First therapeutic drug monitoring of experimental favipiravir in Borna disease virus 1 (BoDV-1) encephalitis patients reveals significant gaps in antiviral treatment: a pilot investigation

**DOI:** 10.1186/s40001-025-03117-x

**Published:** 2025-10-04

**Authors:** Michael Paal, Katharina Habler, Alice Ewert, Michael Vogeser, Leonie Grosse, Victoria Lieftüchter, Simone C. Tauber, Johannes Schiefer, Petra Allartz, Dennis Tappe, Kirsten Pörtner

**Affiliations:** 1 Institute of Laboratory Medicine, Ludwig-Maximilians-University Hospital Munich, Munich, Germany; 2 Department of Pediatrics, Dr. Von Hauner Children’s Hospital, Ludwig-Maximilians-University Hospital Munich, Munich, Germany; 3 Center for Children with Medical Complexity – iSPZ Hauner, Ludwig-Maximilians-University Hospital Munich, Munich, Germany; 4 Department of Neurology, RWTH Aachen University Hospital, Aachen, Germany; 5 Reference Laboratory for Bornaviruses, Bernhard Nocht Institute for Tropical Medicine, Hamburg, Germany; 6 Department of Infectious Disease Epidemiology, Robert Koch Institute, Berlin, Germany

**Keywords:** Borna disease virus 1 (BoDV-1), Encephalitis, Favipiravir (FPV), Therapeutic drug monitoring (TDM), Liquid chromatography tandem mass spectroscopy (LC–MS/MS)

## Abstract

**Background:**

The Borna disease virus 1 (BoDV-1) causes a rare, but severe form of encephalitis in Germany, characterized by rapid progression, late diagnosis, and a high case fatality. Therapy is experimental and recommendations are lacking. Favipiravir (FPV) suppresses BoDV-1 replication in vitro and has been used in a handful of BoDV-1 patients within individual treatment attempts, but little is known about the drug´s pharmacokinetics in encephalitis.

**Methods:**

To monitor and therefore optimize experimental FPV treatment, we established a liquid chromatography tandem mass spectroscopy (LC–MS/MS) assay for serum and cerebrospinal fluid (CSF), and analyzed stored specimens of two patients with BoDV-1 encephalitis in the context of therapeutic drug monitoring as a pilot investigation.

**Results:**

We demonstrate for the first time that orally administered FPV reaches the CSF in BoDV-1 encephalitis. However, the half-maximal inhibitory concentration (IC50) for BoDV-1 was met only in one patient, raising questions on significantly higher dosing and/or alternative formulations for effective treatment.

**Conclusion:**

In conclusion, monitoring experimental FPV therapy in BoDV-1 encephalitis is feasible and should be performed continuously. Future prospective in-depth (multicenter) studies should include more patients and focus on dose-finding, dose–response relationships, and define a therapeutic index to improve outcomes of this so far nearly uniformly fatal disease.

## Background

The Borna disease virus 1 (BoDV-1; species *Orthobornavirus bornaense*) is known since 2018 as the causal agent of rare, but typically fatal zoonotic encephalitis in humans [1, 2]. As of early 2025, 50 (sporadic) BoDV-1 encephalitis cases with molecular confirmation are notified to German health authorities, with many of them retrospectively identified; cases date back to 1992. The annual incidence comprises 5–10 infections, seen in different hospitals across the country, with only one incident case in 2024 and five cases in 2023. Human infections have exclusively been identified in Germany so far, even though the endemic region (known from animal cases) extends to parts of the neighboring countries Switzerland, Austria, and Liechtenstein [3]. All but one (notified, not published) molecularly confirmed BoDV-1 case had a fulminant disease course of encephalitis with lethal outcome (source: Robert Koch Institute) and there is no evidence for a clinical presentation other than encephalitis [4, 5]. Diagnosis is challenging since characteristic features on neuroimaging and demonstrated seroconversion typically appear late [6, 7]; viral detection in cerebrospinal fluid (CSF) is often unsuccessful [7]. Besides unknown transmission routes and a very rapid disease progression, the high case fatality is characteristic of BoDV-1 encephalitis leaving a narrow time window for diagnosis and treatment attempts [8, 9]. BoDV-1 establishes persistent infections intranuclearly [10], and therefore probably requires an extended, if not life-long antiviral therapy [11]. The virus is non-cytopathogenic and inflammation and tissue destruction is immunopathologically induced, requiring additional immunosuppressive therapy as suggested recently [11–13]. However, up to now treatment recommendations for BoDV-1 encephalitis are lacking, and individual antiviral treatment attempts are based on *in vitro* data only.

*In vitro,* the most promising antiviral substance is favipiravir (FPV, T-705; 6-fluoro-3-hydroxypyrazine-2-carboxamide) [14]. FPV is a broad-spectrum inhibitor of viral RNA polymerase with a shelf life of 60 months, that is licensed for the treatment of novel influenza in Japan [15]. Available in an oral formulation only, it has been used as (experimental) treatment of for example Ebola virus disease [16], Lassa fever [17], and COVID-19 [18]. The drug’s safety profile is well established, making it interesting for experimental treatment of emerging viral infections [19, 20]. In vitro, FPV suppresses BoDV-1 transcription and replication in a dose- and time-dependent manner; the half-maximal inhibitory concentration (IC50) for BoDV-1 is 319 ± 99 µM, equivalent to 50.1 ± 15.6 µg/mL [14]. The pharmacokinetics of FPV are well characterized for the treatment of influenza [21], but little is known on the pharmacokinetics in CSF, in the BoDV-1 infected patient, or in the critically ill patient [22], the typical condition of BoDV-1 patients by the time of diagnosis [9]. As a prodrug, FPV with a bioavailability of almost 100% becomes activated after intracellular ribosylation and phosphorylation. FPV undergoes extensive hepatic metabolism, with the metabolites primarily excreted in the urine. BoDV-1 encephalitis therapy with FPV is currently experimental, the dosing and efficacy are unknown and clinical data are limited to single treatment attempts where FPV was given as a salvage therapy. A recent case series analyzed that in the published six (confirmed) BoDV-1 encephalitis cases treated with FPV to date, the drug was administered as late as 7 days before death, far too late to demonstrate any clinical benefit or even prevent fatal outcomes [9]. Moreover, it remains unclear whether the standard oral dosage achieves sufficient concentrations of the drug against BoDV-1 at the site of infection, the central nervous system, in the critically ill encephalitis patient at all. Therefore, knowing such basic pharmacokinetics of FPV in BoDV-1 encephalitis is crucial to further evaluate the drug’s potential therapeutic benefit.

Several methods for the therapeutic drug monitoring (TDM) of FPV have been described before [23–30]. However, these assays are designed exclusively for TDM in blood, and their calibration range is tailored to the lower FPV half-maximal effective concentration (EC50) for SARS-CoV-2, which is 61.88 µM [31], corresponding to 9.72 µg/mL.

The objective of the present investigation was to pilot a first therapeutic drug monitoring for FPV in BoDV-1 encephalitis to possibly evaluate CSF concentrations in the context of an individual treatment attempt and therefore to better monitor and even optimize future FPV therapy strategies. In a first step, a robust assay for quantification of FPV in CSF had to be established.

## Patients, materials, and methods

### Patients and samples

From two BoDV-1 encephalitis cases treated in intensive care units in different university hospitals in Germany in 2022 (termed here Case 1 and Case 2, respectively), a total of 20 archived CSF samples and 17 archived serum samples (all stored at −80 °C) were used. From Case 1, an almost 7-year-old patient (bodyweight 23 kg) with PCR-confirmed BoDV-1 encephalitis [11], six CSF and five serum samples were available for TDM. From Case 2, a 18-year-old patient (bodyweight ca. 80 kg) with positive indirect immunofluorescence antibody test and immunoblot results for BoDV-1 infection [32], 14 CSF, and 12 serum samples were employed in TDM. Samples had been stored at −80 °C for around 24 months. The diagnosis of BoDV-1 encephalitis in this case was strongly supported by clinical, laboratory, radiological, differential diagnostic, and epidemiological findings even though this patient was classified as a probable BoDV-1 encephalitis case according to published case definitions [33]. Formal confirmation of the diagnosis was pending as brain samples were not available and repeated molecular diagnostics in CSF had been unsuccessful.

Additional analyses, including clinical chemical parameters for evaluating liver function and the integrity of the blood–brain barrier were retrieved from the individual patient’s clinical files; in clinical practice, the albumin CSF/serum ratio and CSF white blood cell count serve as markers to assess the blood–brain barrier permeability and the extent of meningeal inflammatory response, respectively. These (previously unpublished) clinical parameters had been measured using standard clinical chemistry assays during their respective hospital stays.

After differing loading doses (Case 1: 2400 mg on day 0, Case 2: 2200 mg on day 0 and 1), both cases were treated with the same oral standard dosage of FPV comprising 1200 mg/d (administered in two single doses of 600 mg each) (influenza dosing day 0: 3200 mg, days 1–5: 1200 mg) for a total period of (i) Case 1 43 days and (ii) Case 2 91 days including a break of 35 days administered via a nasogastric tube. The two cases had differing additional immunosuppressive medication and many other accompanying drugs, such as opioids, antibiotics, anticonvulsants, and sedatives. Treatment with FPV was administered in the context of an individual treatment attempt according to the Declaration of Helsinki, Article 37. The clinical presentation, therapy attempts, and the course of disease are detailed elsewhere [11, 32].

### TDM set-up

Before the TDM of FPV could be performed, a liquid chromatography tandem mass spectroscopy (LC–MS/MS) assay had to be established in the range of 1.00–100 µg/mL, requiring only 25 µL of sample volume, and using isotope-labeled [^13^C_1_-^15^N_1_]-FPV for internal standardization. Following sample cleanup with the addition of 100 µL precipitation reagent (pure methanol containing 1.25 µg/mL [^13^C_1_-^15^N_1_]-FPV), the supernatant was diluted 1:51 with water–methanol (75/25; v/v). A 3µL aliquot was then injected into a Waters Xevo TQ-S–MS/MS system operating in ESI + mode for multiple reaction monitoring, using the transitions 158.1 > 140.9 (quantifier) and 158.1 > 113.0 (qualifier) for FPV, and 160.0 > 113 for [^13^C_1_-^15^N_1_]-FPV. Chromatographic separation was performed on a Raptor Biphenyl Column (2.1 × 100 mm, 2.3 µm particle size, Restek, Bad Homburg, Germany) at 40 °C, with a flow rate of 0.4 mL/min. The mobile phase consisted of solvent A, water–formic acid (99.9/0.1, v/v), and solvent B, methanol–formic acid (99.9/0.1, v/v). The method was validated following the European Medicines Agency (EMA) bioanalytical method validation guideline [34]. Matrix mixing experiments were performed to evaluate whether serum-based calibrators could be reliably used for FPV quantification in CSF.

In a second step, the validated LC–MS/MS method was applied to stored serum and CSF samples from the two BoDV-1 encephalitis cases.

## Results

### Establishment of TDM

**Fig. 1:**
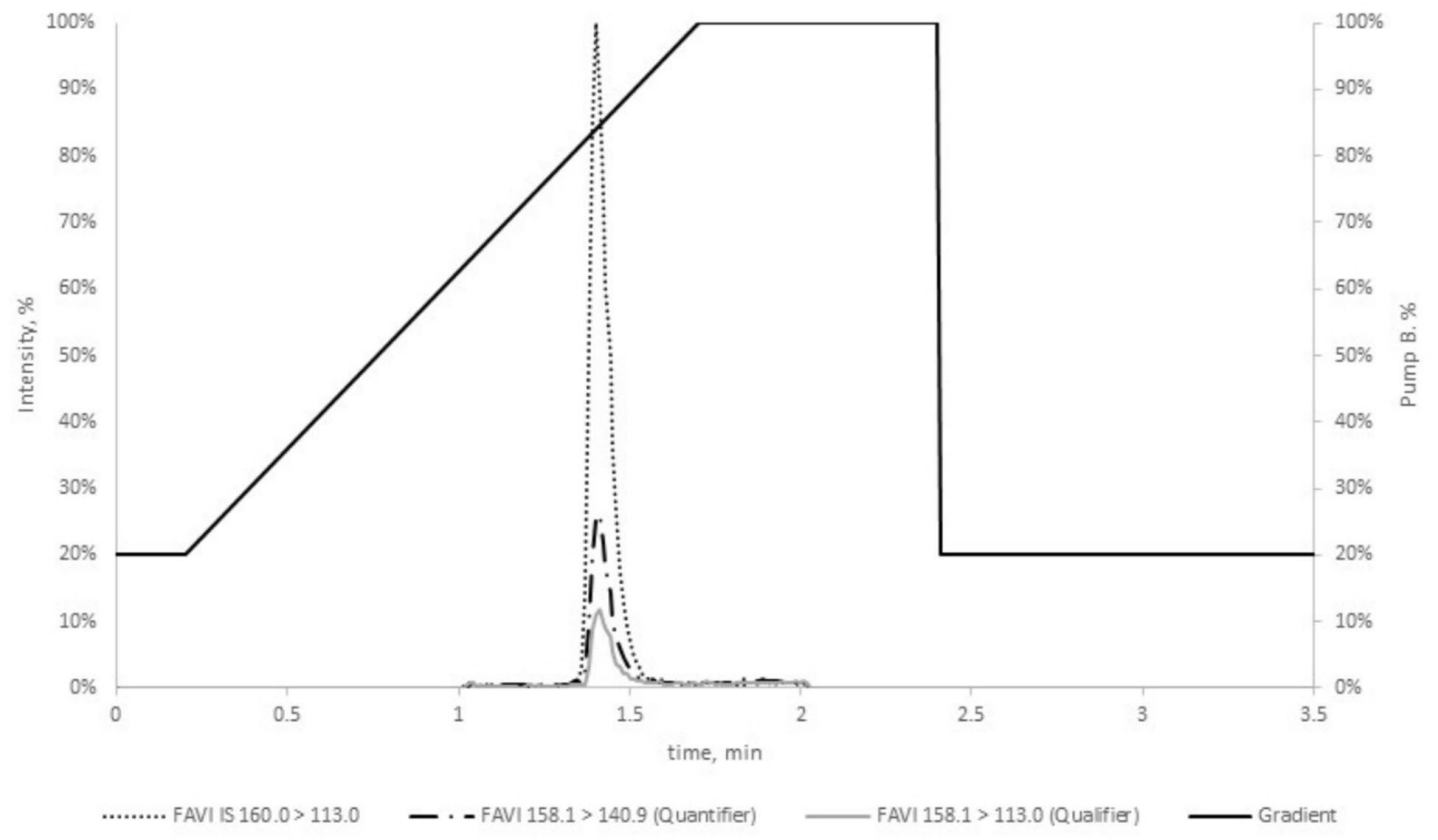
Representative total ion chromatogram of calibrator level 2 with the corresponding chromatographic gradient. Solvent A, water–formic acid (99.9/0.1, v/v); solvent B, methanol–formic acid (99.9/0.1, v/v). Mobile phases were delivered with a flow rate of 0.40 mL/min at 40 °C. *FAVI* favipiravir, *min* minute

Calibration for FPV was conducted using six calibrators (1.00, 2.00, 8.00, 20.0, 50.0, 100 µg/mL) employing a linear regression model with a weighting factor 1/X, excluding the origin. The method demonstrated linearity across all measurement series with a coefficient of determination (*R*^2^) ≥ 0.998. All calibrators, including the lower limit of quantification (LLOQ), adhered to the EMA guideline specifications, with deviations from nominal concentrations ≤ 11.2%. The analyte signal-to-noise ratio (S/N) at the LLOQ consistently exceeded the minimum required value of five, with S/N ≥ 180. A representative LC–MS/MS chromatogram for calibrator level 2 and the chromatographic gradient is shown in Fig. 1.

Intra- and inter-day inaccuracies ranged from −6.0% to 0.38%, while imprecision, measured as the coefficient of variation (CV), remained ≤ 11.4% for all quality controls (1.00, 2.50, 10.0, 40.0, 80.0 µg/mL) and therefore within the ± 15% limits outlined by the EMA. Carry-over, defined as the percentage of peak areas in blank samples injected after the highest calibrator, was < 1.0% for both FPV and its internal standard, meeting the EMA requirements (≤ 20% of the LLOQ for analytes and ≤ 5% for internal standards). Peak areas of FPV and [^13^C_1_-^15^N_1_]-FPV in 20 blank, anonymized samples from patients on polypharmacy without FPV treatment were < 1%, again fulfilling the EMA selectivity requirements (≤ 20% of the LLOQ for analytes and ≤ 5% for internal standard). The FPV ion ratio (quantifier to qualifier) in each positive sample was 2.20, with deviations consistently ≤ 20% compared to the mean ion ratio of corresponding calibrators. Stability testing using quality controls confirmed that samples could be stored for at least 30 months at −80 °C and up to 14 days at room temperature, with deviations of ≤ 15% from the nominal concentration, as per EMA requirements. Processed samples also remained stable in the autosampler at 10 °C, with deviations ≤ 6.3% from nominal concentrations. Matrix mixing experiments with blank serum and CSF specimens and sample cleanup indicated no significant quantitative matrix effect, with a CV of ≤ 15% as specified by the EMA, allowing serum calibrators to be used for the TDM of both specimen types.

### Clinical course

Clinically, Case 1 was transferred to university hospital 3 days after initial admission, FPV was started the same day (day 0) in the critical ill patient and continued for 43 days. After initial significant improvement, his condition progressively deteriorated, leading to his death 55 days after transferal. Case 2 was intubated on day 4 after hospitalization, FPV was started right after BoDV-1 antibody detection 26 days after initial hospital admission but had to be stopped after 56 days due to cardiac arrest and was afterwards reintroduced for another 19 days before final withdrawal. Despite psychiatric complications and further life-threatening complications in the clinical course with re-admittance to hospital, the patient was finally discharged 73 days after first admission under immunosuppressive drugs for rehabilitation, awake and responsive, weaned from mechanical ventilation but with severe neurological restrictions. As of mid-2025, he is alive, living at home and slowly improving clinically under continued immunosuppressive therapy.

### Blood–brain barrier function

Laboratory analyses showed CSF pleocytosis and blood–brain barrier dysfunction in both patients during their course of illness. In Case 1, pleocytosis was moderate with up to 200 cells/µL over time (normal < 5) with a slight blood–brain barrier disruption (CSF/serum albumin ratio up to 7.78 × 10E^−3^, normal < 4.50 (age adjusted)) and prominent intrathecal IgG immunoglobulin synthesis (max. 78%; Table 1). Case 2 presented with similar CSF laboratory findings, including also a moderate lymphocytic increase in CSF cell count (max. 186/µL over time, normal < 5), a slight disruption of the blood–brain barrier (CSF/serum albumin ratio of up to 8.32 × 10E^−3^, normal < 5.30 (age adjusted)), and prominent intrathecal IgG synthesis (up to 85%; Table 2).

**Table 1:** Results of favipiravir (FPV) therapeutic drug monitoring of Case 1

Days after hospitalization	Material	FPV dosage	FPV concentration (µg/mL)	FPV CSF/serum ratio	CSF/serum albumin ratio x10E^−3^ (age adjusted for 7 years, normal < 4.50)
0 days	Serum	None	< 1.00	N/A	N/A
0 days	Serum	None	< 1.00	N/A	N/A
0 days	Serum	1200 mg/d	35.5*	N/A	N/A
2 days	CSF	1200 mg/d	17.1	N/A	7.78 (no intrathecal IgG production)
5 days	Serum	1200 mg/d	40.1*	0.663	5.50 (no intrathecal IgG production)
5 days	CSF	1200 mg/d	26.6		
6 days	CSF	1200 mg/d	21.7	N/A	7.38 (no intrathecal IgG production)
19 days	CSF	1200 mg/d	52.9*	N/A	N/A
22 days	Serum	1200 mg/d	> 100 (102)*	N/A	N/A
25 days	Serum	1200 mg/d	> 100 (115)*	0.243	2.70 (positive intrathecal IgG 70%)
25 days	CSF	1200 mg/d	27.9		
29 days	Serum	1200 mg/d	80.4*	0.668	N/A
29 days	CSF	1200 mg/d	53.7*		N/A
39 days	CSF	1200 mg/d	N/A	N/A	3.44 (positive intrathecal IgG 78%)
41 days	Serum	1200 mg/d	69.0*	N/A	N/A
43 days	Serum	600 mg/d	< 1.00	N/A	N/A

*CSF* cerebrospinal fluid, *FPV* favipiravir, *N/A* sample/s not available. *IC50 (50.1 ± 15.6 (34.5–65.7 µg/mL)) is reached

**Table 2:** Results of favipiravir (FPV) therapeutic drug monitoring of Case 2

Days after hospitalization	Material	FPV dosage	FPV concentration** (µg/mL)	FPV CSF/serum ratio	CSF/serum albumin ratio x10E^−3^ (age adjusted for 18 years, normal < 5.30)
10 days	Serum	None	< 1.00	N/A	N/A
10 days	CSF	None	< 1.00	N/A	N/A
15 days	CSF	None	< 1.00	N/A	N/A
21 days	Serum	2200 mg/d	2.49	1.55	N/A
21 days	CSF	2200 mg/d	3.85		N/A
29 days	Serum	1200 mg/d	3.28	0.692	N/A
29 days	CSF	1200 mg/d	2.27		N/A
41 days	Serum	1200 mg/d	13.5	< 0.0741	7.17 (3-class-reaction, positive intrathecal IgG ca. 85%)
41 days	CSF	1200 mg/d	< 1.00		
49 days	Serum	1200 mg/d	1.26	1.51	5.05 (3-class-reaction, positive intrathecal IgG ca. 85%)
49 days	CSF	1200 mg/d	1.90		
68 days	Serum	1200 mg/d	14.7	0.178	N/A
68 days	CSF	1200 mg/d	2.61		N/A
98 days	Serum	1200 mg/d	< 1.00	N/A	N/A
98 days	CSF	1200 mg/d	< 1.00	N/A	N/A
105 days	Serum	1200 mg/d	23.6	0.534	8.32 (3-class-reaction, positive intrathecal IgG ca. 40%)
105 days	CSF	1200 mg/d	12.6		
138 days	CSF	None	2.59	N/A	N/A
138 days	Serum	None	< 1.00	N/A	N/A
138 days	CSF	None	< 1.00	N/A	N/A
138 days	CSF	None	< 1.00	N/A	N/A
138 days	Serum	None	< 1.00	N/A	N/A
138 days	CSF	None	< 1.00	N/A	N/A
399 days	Serum	None	< 1.00	N/A	N/A
399 days	CSF	None	< 1.00	N/A	N/A

*CSF* cerebrospinal fluid, *FPV* favipiravir, *N/A* sample/s not available. **IC50 (50.1 ± 15.6 (34.5–65.7 µg/mL) is never reached in this patient

### Therapeutic drug monitoring of favipiravir

With the new TDM assay, FPV could be detected in serum and CSF of both patients in different concentrations over time (Tables 1 and 2) with a CSF/serum ratio of up to 0.668 (Case 1) and maximal 1.55 (Case 2). In Case 1, FPV concentrations exceeded the IC50 levels at different time points, while in Case 2 concentrations barely reached the measuring range in CSF, never reaching the IC50 levels. Laboratory tests and sonography in both cases indicated no significant impairment of the liver function, a distinct protein deficiency, or fluid disbalance that might have affected the distribution or/and metabolism of FPV.

## Discussion and conclusion

In our retrospective pilot investigation, we successfully assessed antiviral drug levels in the CSF of two BoDV-1 encephalitis cases with divergent outcomes. Our analyses reveal substantial FPV drug concentrations in CSF for the first time, underscoring that FPV can reach the site of infection in BoDV-1 encephalitis. However, our results raise questions on drug underexposure when using a standard dosing scheme, even if we demonstrate that the standard oral dosage of FPV can reach CSF concentrations exceeding the IC50 at least in the (light weighted) pediatric case.

In Case 1, significant FPV concentrations were detected in the CSF alongside pathological CSF markers, whereas this was not observed in the likely underdosed Case 2. In Case 1, a correlation of the CSF/serum ratio with the disruption of the blood–brain barrier may be suggested by our limited data; however, only two time points with both data of the blood–brain barrier function were available. In contrast, in Case 2 no such correlation was discernable. Although the pilot character of our investigation hinders direct comparison of the two cases analyzed here, the adult Case 2 showed considerably lower FPV concentrations than the pediatric Case 1, both in serum and CSF. As Case 2 received the same standard (influenza) non-weight adjusted dosage like Case 1, but weighed much more than Case 1, a relative underdosing seems probable. However, individual differences in FPV absorption, tissue penetration, diffusion, or distribution in this patient due to co-medication, co-morbidities, or the severity of disease might also be possible explanations. Independent of our results and besides the dosage approved for the treatment of influenza virus (day 1: 3200 mg, days 2–5: 1200 mg) with an IC50 of 0.013 to 0.48 μg/mL [35], the JIKI-trial for the treatment of Ebola virus with a considerably higher IC50 of 10 µg/mL [36] used FPV in double doses (day 1: 6000 mg; day 2 to day 10: 2400 mg/d) [16] and showed that the drug concentrations aimed for were still not reached [21]. Interestingly, BoDV-1 has an IC50 up to six times higher than that of Ebola virus. This suggests that in BoDV-1 encephalitis patients, FPV needs to be dosed significantly higher than in the treatment of influenza or Ebola virus disease to reach concentrations stably exceeding the IC50, which is needed to sustainably suppress viral replication. Of note, lower FPV concentrations (in serum) in the critically ill patient (suffering from COVID-19 compared to healthy subjects) have been described before, suggesting that the bioavailability of FPV differs depending on the severity of illness [22]. Like the few other published BoDV-1 patients treated with FPV so far (*n* = 5) [9], both of our cases were critically ill and mechanically ventilated by the time of FPV initiation. Owing to the severity of disease, both of them might have needed higher FPV doses. However, FPV was stopped in Case 2 after the development of significant bradycardia mounting in cardiac arrest. Bradycardia or other electrocardiographic effects have been considered a possible side effect of FPV during the treatment of other infections before [17, 37–39], and it is therefore highly questionable if Case 2 would have tolerated higher doses of FPV anyway. However, in the JIKI-trial evaluating safety and effectiveness of FPV in 111 patients with Ebola virus disease, high doses of FPV were well tolerated clinically and paraclinically, and no grade 3 or 4 clinical events were considered to be drug related [16]. Other trials or case reports on Ebola virus disease using high-dose FPV could also not confirm the concerns regarding potential general cardiotoxicity (reviewed in [40]). Further studies concerning safety of high-dose FPV are therefore certainly needed, not only to improve outcomes of BoDV-1 encephalitis. However, given the poor prognosis of untreated BoDV-1 encephalitis, safety concerns of salvage treatment almost take a back seat. Even though our call for higher dosing requires safety considerations, they should not be the first argument to suspend the evaluation of high-dosed FPV in BoDV-1 or viral hemorrhagic fevers [40]. In summary, the standard oral (influenza) dosage likely seems insufficient for achieving therapeutic levels in adult BoDV-1 patients and underlines the urgent need for continuous and sophisticated TDM using the robust assay we developed. Besides higher dosing and alternative (parenteral) administration routes, FPV probably needs to be initiated early in the disease course to show any potential clinical benefit [9]. However, developing parenteral FPV formulations requires not only the manufacturers interest but also sophisticated pharmaceutical technology, detailed preclinical testing and in the end also licensing for a given indication. Luckily, an intravenous formulation is supposed to be under development [17].

The reason for the survival of Case 2 despite questionable antiviral drug efficacy and final withdrawal of FPV remains unclear. Survival in this case is possibly independent of the administered FPV treatment, as seen in one other, molecularly confirmed (retrospectively diagnosed) case (Source: Robert Koch Institute), and might—besides other, unknown factors—depend on concomitant iatrogenic immunosuppression to quell virus-induced inflammation, individual host factors (for example medical complications, age, susceptibility to the virus) and disease severity [12, 13]. The reason for the death of Case 1 despite higher FPV levels, early diagnosis and treatment initiation, and initial clinical improvement remain just as unclear. An underlying immunodeficiency was excluded in this case by whole exome analysis. Whether the detected herpes simplex virus in CSF or the low (immunosuppressive) ciclosporin A plasma levels measured in the disease course contributed to the unfortunate outcome remains unanswered [11]. The causal link between our dosing observation and the clinical outcome in the end is unclear. The underlying pathomechanisms of BoDV-1 encephalitis are ultimately not fully understood. Immunologically mediated cerebral tissue destruction seems to determine the course of disease and leads to death in the vast majority of cases. Survival independent of antiviral treatment so far is a very rare event and the absolute exception, making it difficult to understand the underlying reasons.

Limitations of our study are the small sample size on one hand and the retrospective nature of the study on the other, hindering us to fully characterize the pharmacokinetics of FPV in BoDV-1 encephalitis: Our results—even if they are pioneering work—offer only an initial but important glimpse, limiting generalizability of the measured drug levels so far. Further prospective studies with a larger sample size and standardized sampling protocols including timed collection and detailed clinical metadata (such as co-medications, liver and kidney functions, etc.) are necessary to fully elucidate FPV pharmacokinetics such as AUC calculations, time-course modeling, and penetration rates in BoDV-1 encephalitis. Given the low incidence, however, the recruitment will likely take years. A strong pre-formed collaboration between treating hospitals and diagnosing and analyzing laboratories, as well as health authorities, will be needed to further understand the course of disease and potential causal therapies.

In conclusion, we demonstrate that TDM of FPV in CSF and serum of BoDV-1 encephalitis patients is not only well feasible but urgently needed. Our results suggest that higher oral doses in adult patients or alternative, parenteral administration routes have to be generally considered in BoDV-1 encephalitis treatment attempts. Our analyses also underscore the need for research on BoDV-1 encephalitis treatment with FPV in order to establish clear dosing recommendations and maximize treatment effects. Pharmacokinetic analyses, including penetration across the blood-brain barrier, pharmacokinetic modeling, biologic response (e.g., EC50), and probability of target attainment studies, are needed in a large patient cohort with standardized protocols. However, the rareness of the disease with an estimated total of 5–10 incident BoDV-1 encephalitis cases per year exclusively identified in Germany so far, besides often unsuccessful BoDV-1 RNA detection in CSF during the course of disease, casts doubt on the feasibility of powerful, interinstitutional clinical trials. Nonetheless, dose-finding studies in healthy volunteers are key to assess tolerance and concentrations with high doses [21]. As drug concentrations in CSF likely (also) depend on the integrity of the blood–brain barrier, patients suffering from encephalitis should also be investigated systematically. These insights are crucial in refining antiviral strategies for BoDV-1 encephalitis to finally improve patient outcomes in the long run. However, antiviral therapy is just one facet of a comprehensive, multimodal treatment strategy for BoDV-1 encephalitis likely also needing distinct clinical awareness, prudent immunosuppression, and strong supportive care.

## Data Availability

All relevant data are within the paper.

## Abbreviations

BoDV-1: Borna disease virus 1
FPV: Favipiravir
CSF: Cerebrospinal fluid
TDM: Therapeutic drug monitoring
LC–MS/MS: Liquid chromatography tandem mass spectroscopy
IC50: Half-maximal inhibitory concentration
EC50: Half-maximal effective concentration
EMA: European Medicines Agency
LLOQ: Lower limit of quantification
S/N: Signal-to-noise ratio
CV: Coefficient of variation
AUC: Area under the curve
N/A: Sample/s not available
AMG: Arzneimittelgesetz
